# Despite higher revision rate, MoM large-head THA offers better clinical scores than HR: 14-year results from a randomized controlled trial involving 48 patients

**DOI:** 10.1186/s12891-021-04286-6

**Published:** 2021-04-30

**Authors:** Lazaros Kostretzis, Martin Lavigne, Marc-Olivier Kiss, Maged Shahin, Janie Barry, Pascal-André Vendittoli

**Affiliations:** Surgery Department, Hôpital Maisonneuve-Rosemont, Montreal University, 5415 Boulevard de l’Assomption, Montréal, Québec H1T 2M4 Canada

**Keywords:** Hip resurfacing, Large diameter, Metal ions, Metal on metal, Patient reported outcome measures, Radiographic, Revision rate, Total hip arthroplasty

## Abstract

**Background:**

The high failure rates of metal on metal (MoM) large diameter head total hip arthroplasty (LDH THA) and hip resurfacing (HR) prevented their long-term comparisons with regards to clinical outcome. Such knowledge would be important as ceramic LDH bearing is now available. With long-term follow-up, we investigated the difference in 1) patient-reported outcome measures (PROMs); 2) revision and adverse events rates, and 3) metal ion levels between MoM LDH THA and HR.

**Methods:**

Forty-eight patients were randomized for LDH THA (24) or HR (24) with the same MoM articulation. At a mean follow-up of 14 years, we compared between groups different PROMs, the number of revisions and adverse events, whole blood Cobalt (Co) and Chromium (Cr) ion levels, and radiographic signs of implant dysfunction.

**Results:**

LDH THA (all cases: revised and well-functioning) had significantly better WOMAC (94 versus 85, *p* = 0.04), and more frequently reported having no limitation (*p* = 0.04). LDH THA revision rate was 20.8% (5/24) versus 8.3% (2/24) for HR (*p* = 0.4). Mean Co and Cr ion levels were higher in LDH THA compared to the HR (Co: 3.8 μg/L vs 1.7 μg/L; p = 0.04 and Cr: 1.9 μg/L vs 1.4 μg/L, *p* = 0.1). On radiographic analyses, 2 LDH THAs showed signs of adverse reaction to metal debris, whereas 1 loose femoral HR component was documented.

**Conclusion:**

In the long-term, MoM LDH THA had a high trunnion related revision rate but nonetheless showed better PROMs compared to HR. Provided with a well-functioning modular junction, non-MoM LDH THA would offer an appealing option.

**Trial registration:**

ClinicalTrials.gov (NCT04516239), August 18, 2020. Retrospectively registered.

## Background

The favorable tribological properties of metal on metal (MoM) bearings promised, in the early 2000s, to reduce polyethylene wear-related osteolysis and improve joint stability [1]. This led to the reintroduction of hip resurfacing (HR) and the development of large diameter head total hip arthroplasty (LDH THA). In 2010, our institution was awarded the John Charnley award for our randomized controlled trial (RCT) comparing MoM HR with MoM LDH THA. In that study, we found similar performance on most functional tests (including formal gait analysis), and similar patient reported outcome measures (PROMs) between groups [2]. Moreover, no significant difference was observed between both groups and a control group without hip replacement. However, the mean follow-up of 14 months was short, and long term follow up may reveal different findings.

Unfortunately, clinical studies and national registries [3, 4] reported unacceptable rates of failure in the mid-term for most MoM HR and LDH THA. leading to recall and voluntary removal of these implants from the market [5–7]. However, design and technological improvements using other materials, like ceramic [8, 9] may have the potential to mitigate the limitations associated with MoM and may lead to resurgence of LDH THA and HR.

The purpose of this study was to compare HR and LDH THA randomized in our study after a mean follow-up of 14 years (12–15) regarding 1) PROMs, 2) revision and adverse events rates, and (3) systemic metal ion levels.

## Materials and methods

### Study design

**Fig. 1:**
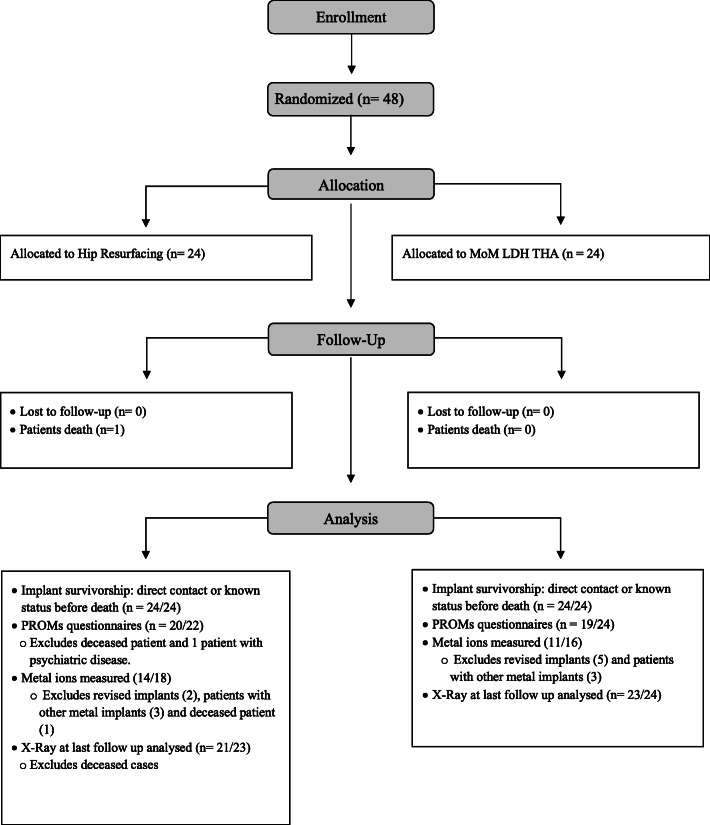
The figure shows the flow chart of participants throughout the study

Between February 2006 and April 2007, 48 patients aged less than 70 years with degenerative hip joint disease were randomized for MoM HR (24) or LDH THA (24, Fig. 1). Patient demographics are presented in Table 1. The same original Durom acetabular cup (not the USA version) was implanted in all patients. A posterior approach was used for both interventions, with the short external rotators released from the greater trochanter and a posterior capsulotomy performed. In the HR group, the capsulotomy was completed circumferentially, the gluteus minimus was elevated from the ilium, and the gluteus maximus tendinous insertion on the femur was released in all men, but only when needed in women. For more details on the implants and surgical technique, a detailed protocol of the this RCT has been published previously [2]. The present study adhered to CONSORT guidelines.

**Table 1:** Pre- and peri-operative data for patients who received the allocated treatment

Factor	HR	LDH THA	***P***
	*n* = 24	*n* = 24	
Gender (male/female)	14/10	15/9	0.9
Primary diagnosis			1
Primary osteoarthritis	18	19	
Protrusio acetabuli	1	1	
Post-traumatic arthritis	1	–	
Hip dysplasia	2	1	
Osteonecrosis	1	2	
Rheumatoid arthritis	1	–	
Post-septic arthritis	–	1	
Age in years at surgery mean (SD)	50 (7.1)	50 (7.8)	0.6
BMI mean (SD)	28 (5.9)	28 (4.1)	1
Acetabular vertical angle mean (SD)	49 (7.5)	43 (8)	0.01
HR femoral component CCD angle mean (SD)	142 (7.7)	–	–
Acetabular cup diameter mean (SD)	54 (3.8)	54 (3.8)	0.9
Bearing diameter, mean (SD)	48 (3.8)	48 (3.8)	0.9

*HR* Hip resurfacing, *LDH THA* Large diameter head total hip arthroplasty, *BMI* Body mass index, *CCD* Cervico diaphyseal angle

### Assessment of outcomes

At last follow-up, different PROMs were collected for patients enrolled in the study (including revised cases): WOMAC [10], UCLA activity score [11], Forgotten Joint Score (FJS-12) [12], and Patient’s Joint Perception (PJP) [13]. All implant revisions and adverse events during the follow-up period were recorded. Whole blood Cobalt (Co) and Chromium (Cr) ion measurements were performed in patients with original hip implants (not revised) and without other bodily metallic implants at the 1 year and last follow-up time frame. All samples were submitted for blinded analysis by an independent laboratory using a Finnigan MAT Element 2 high-resolution sector-field inductively coupled plasma mass spectrophotometer (Thermo Fisher Scientific GmbH, Bremen, Germany). The detection limits were 0.1 μg/L for Cr and 0.01 μg/L for Co. An anteroposterior (AP) radiograph of the pelvis with the legs positioned in 15° of internal rotation and a cross-table lateral radiograph of the operated hip were taken at each follow-up visit and compared with immediate post-operative films by one author (LK) seeking for signs of implant dysfunction. Definite femoral stem loosening was defined by continuous lucent lines > 2 mm, stem fracture, subsidence > 5 mm, or a change in component angulation > 5° [14–16]. Definite acetabular loosening was considered with continuous radiolucency > 2 mm, component migration > 3 mm or angulation change > 5° [17]. Heterotopic ossification grade was estimated according to the Brooker classification [18]. Description of radiolucent lines were done according to Amstutz zones [19] (HR) and Gruen zones [20] (LDH THA) for the femur, and Charnley-De Lee zones [21] for the acetabulum.

### Statistical analysis

Continuous variables are presented as means (SD) and categorical variables as frequencies. For primary and secondary outcomes, groups were compared by Chi-square and Mann-Whitney tests for categorical and continuous variables, respectively. The Mann-Whitney test was used to assess the asymmetric distribution of the continuous variables. Fisher’s exact test was used when the expected frequencies were too low. The effect of time on PROMs and ion levels within the groups (paired samples) was tested with the non-parametric Wilcoxon Signed Ranks Test. The significance level was defined as *p* < 0.05. Statistical analyses were performed with SPSS 25.0 software (SPSS Inc., Chicago, IL, USA).

## Results

Mean follow up for both groups was 14 years (12.1 to 14.7).

### Patient reported outcome measures (PROMs)

**Fig. 2:**
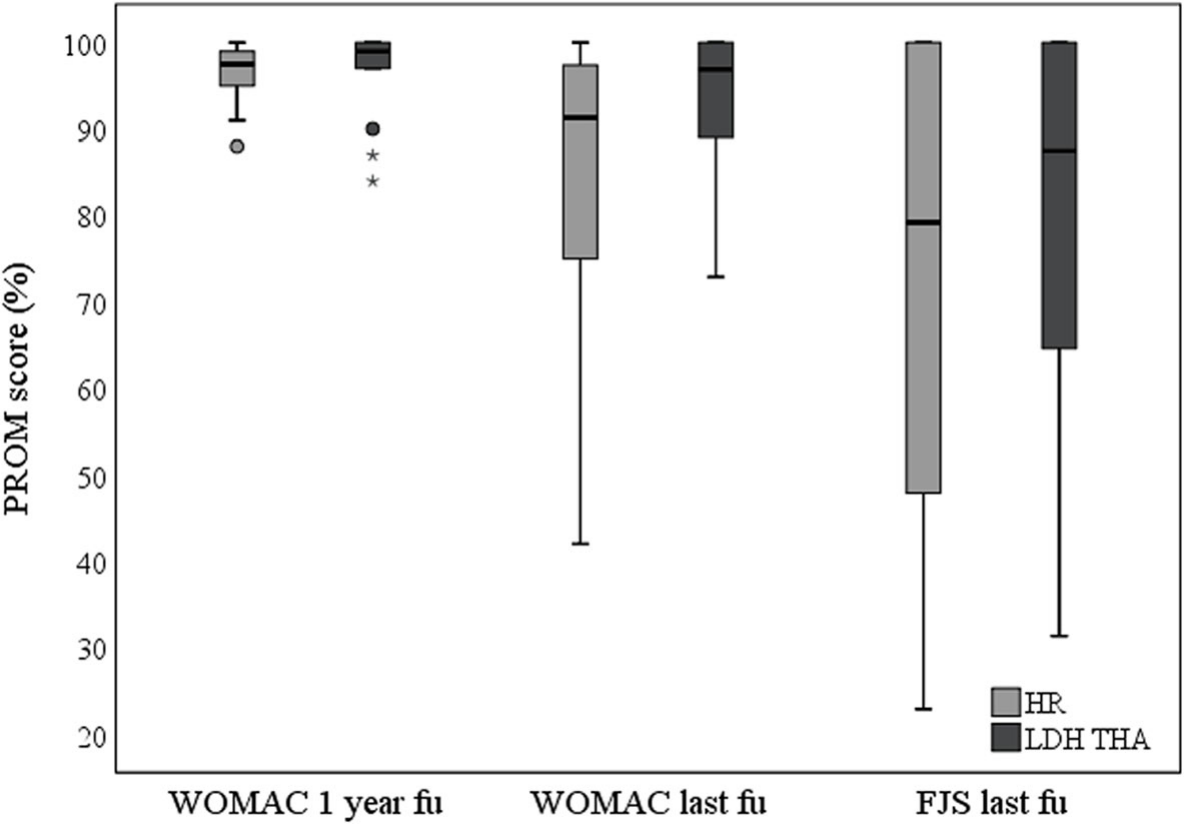
Box plot chart of one-year and last follow-up WOMAC scores, and last follow-up FJS for patients with LDH THA or HR. WOMAC (higher values represent a better outcome). The line at the center of the boxes shows the median value. Box lengths represent the interquartile rage (1st to 3rd quartiles). Data flagged by ° are outliers (being more than 1.5 to 3.0 times the interquartile range over the third quartile), and data indicated by * are extreme values (more than 3 times the interquartile range over the third quartile)

Following an intent to treat analysis, including all cases (including revised cases), last follow-up, mean WOMAC scores were significantly better in the LDH THA group (94 vs 85, *p* = 0.04, Fig. 2, Table 2), and significantly more LDH THA patients reported no limitation with their artificial joints on the PJP question (p = 0.04, Table 2). UCLA activity scores, FJS, and overall PJP results, were also higher for the LDH THA group but the difference did not reach a statistically significant difference (Fig. 2, Table 2). Between the 1-year and last follow-up evaluation, a significant deterioration was observed in both groups for WOMAC (HR, *p* = 0.004; LDH THA, *p* = 0.04). Similarly, UCLA activity scores deteriorated for the same period in both groups, reaching a statistical significance only in the LDH THA group (*p* = 0.01) (Fig. 2, Table 2).

**Table 2:** Patient-reported outcome measures (PROMs) data, values are mean (SD) and n for patient’s joint perception (PJP)

PROMs	One-year follow-up			Last follow-up, all cases^**a**^			Last follow-up, well-functioning cases^**b**^		
	HR (n = 24)	LDH THA (n = 24)	***p***	HR (***n*** = 20)	LDH THA (***n*** = 19)	***p***	HR (***n*** = 17)	LDH THA (***n*** = 12)	***p***
**WOMAC**	97 (8.4)	98 (8.5)	0.2	85 (16)	94 (7.8)	0.04	89 (11)	96 (5.3)	0.05
**UCLA Activity**	8.0 (1.5)	8.3 (1.7)	0.5	7.2 (1.8)	6.7 (1.8)	0.5	7.3 (1.9)	6.4 (1.7)	0.3
**FJS**	–	–	–	80 (27)	80 (21)	0.5	80 (21)	85 (14)	0.7
**PJP**			0.8			0.2*			0.04*
**Natural hip**	15	14		8	13		8	9	
**Artificial hip without limitation**	5	7		4	4		2	3	
**Artificial hip with minimal limitation**	4	3		7	2		7	0	
**Artificial hip with significant limitations**	0	0		1	0		0	0	
**Non-functional hip**	0	0		0	0		0	0	

*HR* Hip resurfacing, *LDH THA* Large diameter head total hip arthroplasty, *FJS* Functional joint score, *PJP* Patient’s joint perception

^a^all cases: non-revised, revised and cases with adverse events are included

^b^well-functioning cases: non-revised and without radiographic signs of dysfunction implants are included

*Regrouping the 4 categories in 2: “hip without limitation” and “hip with limitation”, *p* values are 0.04 for all cases and 0.01 for well-functioning cases in the last follow-up

A significant difference was present within each group between one-year and last follow up evaluations for WOMAC (HR, *p* = 0.004; and LDH THA, *p* = 0.04) for all cases. (Related-Sample Wilcoxon Signed Rank Test)

UCLA activity differences were significant only for the LDH THA group between one-year and last follow-up (HR, *p* = 0.5; and LDH THA, *p* = 0.01) for all cases. (Related-Sample Wilcoxon Signed Rank Test)

WOMAC differences were significant only for the HR group between one-year and last follow-up (HR, *p* = 0.009; and LDH THA, *p* = 0.3) for well-functioning cases. (Related-Sample Wilcoxon Signed Rank Test)

When comparing PROMs of well-functioning implants (non-revised and without radiographic signs of implant dysfunction), LDH THA continues to demonstrate significantly better overall PJP results (*p* = 0.04), while the rest of the scores differences did not reach statistical significance (WOMAC nearly significant with *p* = 0.05). Evaluating the effect of time between the 1-year and last follow-up evaluation, both HR and LDH THA had a significant WOMAC score deterioration (*p* = 0.004) (Fig. 2, Table 2).

### Revision rates

**Fig. 3:**
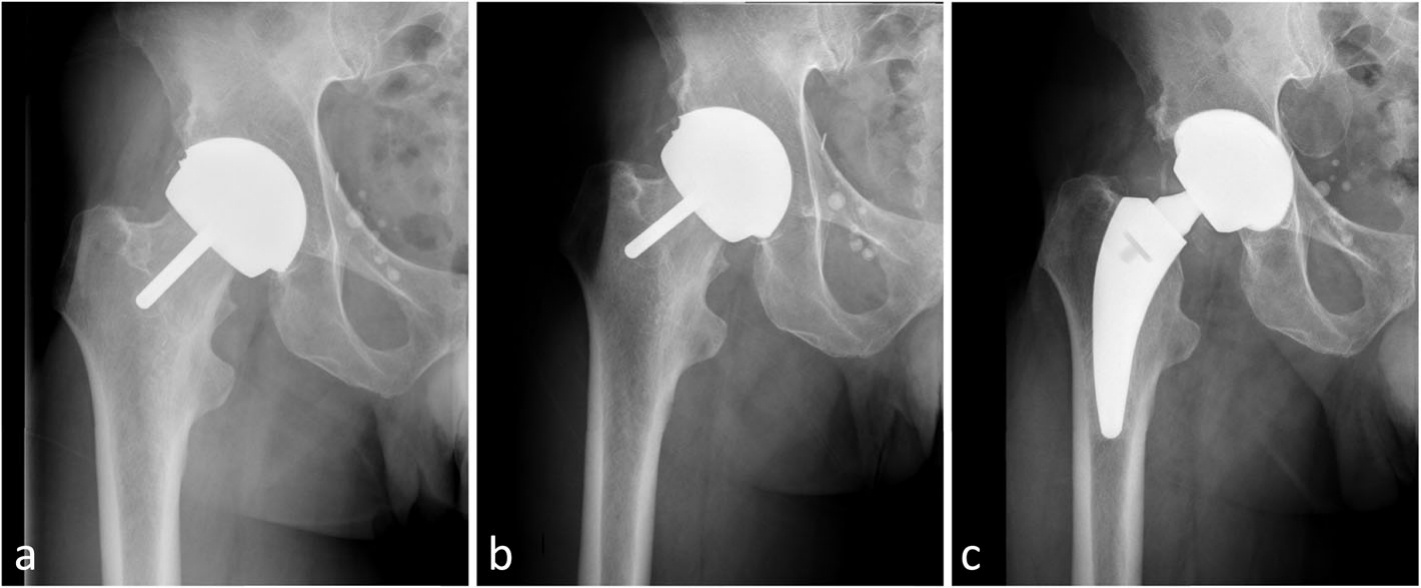
**a** A 54-year-old man with osteoarthritis underwent implantation of hip resurfacing implant. An AP radiograph of the patient’s right hip taken 4 months after surgery. **b** An AP radiograph of the patient’s right hip taken 8 years postoperatively shows the femoral component is loose. **c** The patient underwent bipolar revision with a CoC implant

The overall revision rate was 20.8% (5/24) for LDH THA and 8.3% (2/24) for HR (*p* = 0.4, Fisher’s exact test). In HR, 2 cases of femoral loosening occurred at 2.1 and 8 years after the initial procedure. One case was a woman with primary osteoarthritis, aged 47 years at the time of initial surgery. The bearing size was 44 mm and cup inclination were 42°. The loose femoral component was revised 2 years postoperatively to an uncemented primary stem with a MoM LDH matching the well-fixed acetabular component. Three years later, she developed trunnion related adverse reaction to metal debris (ARMD) and the bearing and the acetabular component were re-revised to a ceramic on ceramic (CoC) articulation, while the stem was retained. The second case was a man with primary osteoarthritis, aged 54 years at the time of surgery. He was implanted with a 48-mm bearing with a cup inclination of 45°. He developed a loose femoral component 8 years postoperatively and had a bipolar revision with a CoC implant (Fig. 3).

**Fig. 4:**
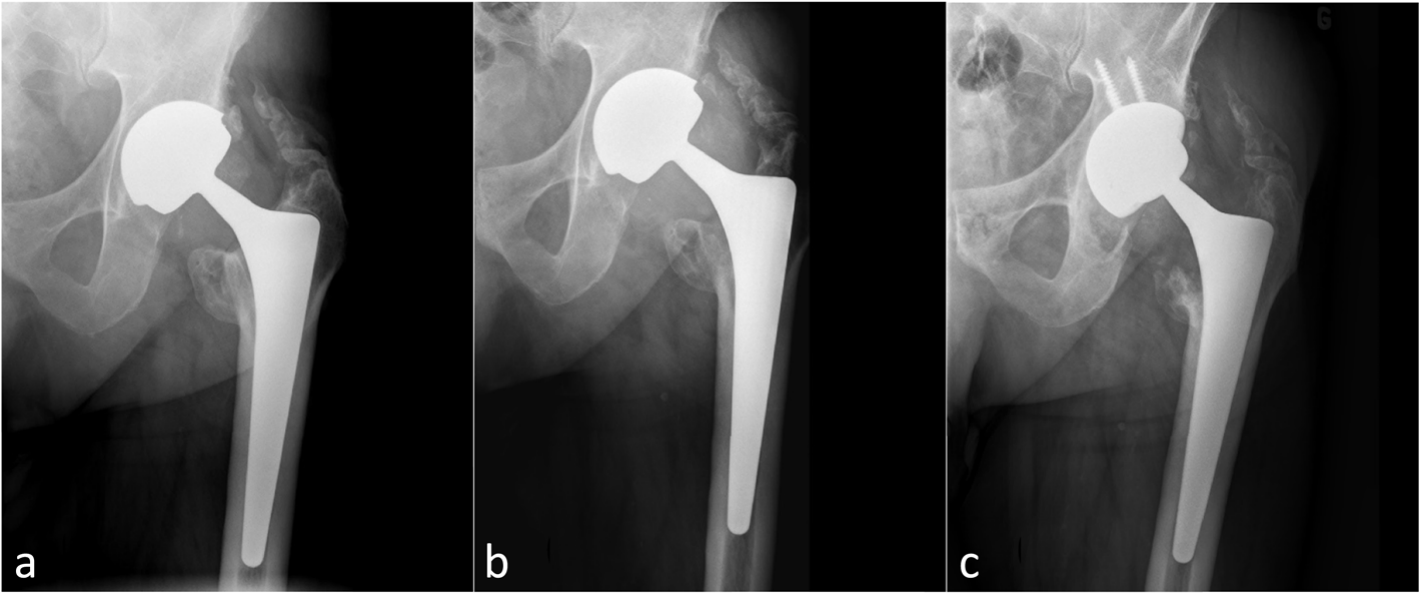
**a** A 63-year-old man with osteoarthritis underwent implantation of large diameter total hip arthroplasty implant. An AP radiograph of the patient’s left hip taken 1 month after surgery. **b** An AP radiograph of the patient’s right hip taken 11 years postoperatively shows proximal femoral lysis. **c** The patient underwent bipolar revision with a CoC implant

In LDH THA, the reasons for revision included 4 ARMD cases and 1 deep infection that was treated by a two-stage revision at 8.8 years after the initial surgery. Revisions for ARMD were performed after a mean of 10.7 (9.3–11.3) years. The revised cases had mean levels of Co and Cr of 2.6 μg/L (1.4 to 4.5) and 1.9 μg/L (1.2 to 3.1), respectively, at 1 year and 2.8 μg/L (0.7 to 4.9) and 1.5 μg/L (1.1 to 2), respectively, before revision. The mean acetabular component abduction angle was 40° (36° to 45°). In all ARMD cases, we observed blackened corroded debris at the junction between the stem and the CoCr femoral head adapter sleeve. During revisions, all acetabular components were revised, and 1 out of the 4 femoral stems was retained; the implanted bearing was a CoC LDH in 3 cases, and metal-on-polyethylene (MoP) in 1 case (Fig. 4). The ARMD revision rate was significantly higher in LDH THA (16.7% versus 0%; *p* = 0.04).

### Other adverse events

During the initial surgery, a hairline proximal femoral fracture occurred in 2 LDH THAs. Both were stabilized with cerclage wires intraoperatively and healed uneventfully with no postoperative restrictions. In HR, 1 patient developed a drop foot and paresthesia immediately postoperatively, that recovered gradually over the course of 2 years. In LDH THA, 1 patient had unexplained mild groin symptoms, fluid collections on MRI, and low and stable ion levels (Co 0.6 μg/L and Cr 1.5 μg/L). ARMD is suspected, but no surgical intervention has occurred to date. The LDH THA patient revised for infection experienced a single episode of dislocation 6 months post revision, that was treated conservatively without recurrence.

### Metal ion levels

**Fig. 5:**
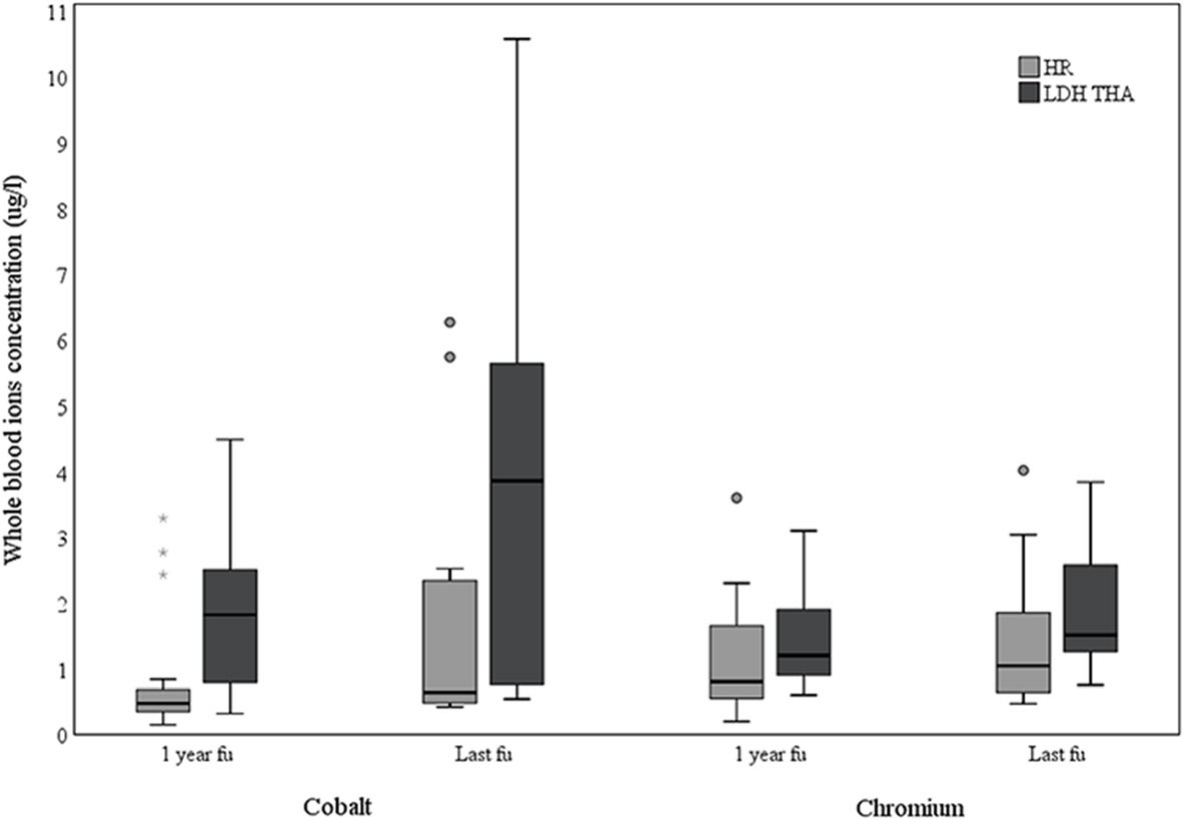
Box plot chart of Cobalt and Chromium ion levels in whole blood of one-year and last follow-up for patients with LDH THA or HR. The line at the center of the boxes shows the median value. Box lengths represent the interquartile rage (1st to 3rd quartiles). Data flagged by ° are outliers (being more than 1.5 to 3.0 times the interquartile range over the third quartile), and data indicated by * are extreme values (more than 3 times the interquartile range over the third quartile)

Co concentrations were significantly higher for LDH THA compared to HR at 1-year and last follow-up (*p* = 0.01, *p* = 0.04, Table 3). Cr levels were higher for LDH THA at 1-year and last follow-up, but statistical significance was not achieved (*p* = 0.2 and *p* = 0.1, respectively). The mean Co/Cr ratio was almost double for LDH THA, compared to HR at 1-year follow-up (1.5 vs 0.8; p = 0.1) and last follow-up (1.9 vs 1.0; *p* = 0.1), but did not reach statistical significance. Over time, Co and Cr levels increased between 1-year and last follow-up for LDH THA (Co *p* = 0.08; Cr *p* = 0.2) and for HR (Co *p* = 0.06; Cr *p* = 0.4) (Fig. 5, Table 3).

**Table 3:** Whole blood Cobalt and Chromium levels at one-year and last follow-up, values (μg/L) are mean (SD)

	One-year follow-up			Last follow-up		
	HR ***n*** = 16	LDH THA ***n*** = 15	***p***	HR ***n*** = 14	LDH THA ***n*** = 11	***p***
**Cobalt**	0.9 (1)	1.9 (1.3)	0.01	1.7 (2)	3.8 (3.2)	0.04
**Chromium**	1.2 (0.9)	1.5 (0.8)	0.2	1.4 (1.1)	1.9 (1)	0.1

*HR* Hip resurfacing, *LDH THA* Large diameter head total hip arthroplasty, Cobalt (HR, *p* = 0.06; and LDH THA *p* = 0.08) and Chromium (HR *p* = 0.4; and LDH THA *p* = 0.2) ion level differences were not significant between the one-year and last follow-up. (Related-Sample Wilcoxon Signed Rank Test)

### Radiological evaluation

At last follow-up, no acetabular component in either group was found to be loose. In HR, 1 femoral component (48 mm in diameter) in a female patient was considered to be loose. Interestingly, multiple cysts in the acetabulum and femoral head were present on the pre-operative radiographs of this patients. This patient remains asymptomatic. Periprosthetic non-progressive (< 2 mm) radiolucent lines were observed in 4/23 of LDH THA cases, in the proximal femur (Gruen zones 1,7, 8 or 14), while no stem was found to be loose. The presence of heterotopic ossification (HO) was similar (*p* = 0.9) in both groups (LDH THA 6/23 vs HR 6/21). None of the patients presenting with HO had any related symptoms. ARMD is suspected in 1 LDH THA patient with persisting groin pain, high metal ion levels (Co 10.1 μg/L, and Cr 2.9 μg/L) and multiple periprosthetic fluid collections on MRI. This patient is awaiting his revision surgery.

## Discussion

The present study documented the long-term follow-up of an RCT comparing LDH THA and HR, both with identical MoM bearing. Assessing PROMS for all cases (including revised ones), LDH THAs provided better WOMAC scores, compared to HR, and patients more frequently reported no limitation with their artificial joints. We found higher revision rates in the LDH THA group and different reasons for revision in each group. The main reasons for revision were femoral head loosening for HRs and ARMD secondary to trunnionosis in LDH THAs. At the last follow-up, Co levels were significantly higher in LDH THA, whereas radiographic outcomes were similar.

### Study limitations

This study has some limitations. First, when the study was designed, gait analysis was the primary outcome and power analysis was calculated accordingly. Interestingly, while this may have limited the power of the study, we could still find statistically significant results, and our data represent the longest findings published from an RCT comparing LDH THA to HR using the same bearing. Second, patients were kept blind to the type of prosthesis implanted for only 1 year after surgery. The impact of their knowledge of implanted components on PROMs after that time point is unknown. Third, patients were not systematically assessed by imaging techniques (ultrasound, MRI) to identify ARMD. It is likely that asymptomatic ARMDs were missed.

### PROMs

In our study, all PROMs were higher in the LDH THA group, even though only WOMAC and the perception of no limitation on the PJP question reached a statistical significance (Table 2). In the LDH THA group, 1/19 patients had a WOMAC score < 80, compared to 6/20 in HR. When comparing WOMAC scores over time for all cases, we found a deterioration for both study cohorts (Table 2, Fig. 2). This may be explained by patients aging, or by the impact of surgical revision on the clinical scores. Interestingly, when we compared PROMs of patients with well-functioning implants, LDH THA was found to have better overall PJP results, but no significant differences in other PROMs (WOMAC was nearly significant with *p* = 0.05). However, there are some limitations to most validated PROMs and patients may feel their hip as “natural” without necessarily having better WOMAC scores [22]. The data from our current study do not provide a robust explanation for the differences in PROMs between groups in the long term. On the other hand, in the senior author’s practice, when revising a HR into a LDH THA, most patients show preference to the revised hip. Similar subjective feedback is given by patients with a HR on one side and subsequently operated on the other side with a LDH THA. They all prefer the LDH THA side, mainly for its increase flexibility and greater range of motion. At a shorter follow up of 9 years, a similar RCT by Konan et al. [23], comparing 104 Durom HR or LDH THA, noted no difference in PROMs. Using a CoC LDH THA in 276 patients, our group, using the same surgical technique as in the current study, observed mean WOMAC score of 92.3, UCLA activity score of 6.6, and FJS of 88.5 after a mean follow-up of 67 months [9]. These results are very similar to the well-functioning MoM LDH THA data of the current study (Table 2).

### Revision rates and other adverse events

In our study, the reasons for revision were implant specific. All HR’s revisions and failed implant on radiographic analysis were due to femoral component loosening (3/24). The failed femoral head diameters were all ≤48 mm, which is considered an important risk factor for loosening [2, 12, 24]. Indeed, Durom HR was reported to have a higher rate of femoral head loosening compared to other HR implants and the US acetabular cup version (not used in the present study) was recalled by the manufacturer in 2008. In the LDH THA group, all revisions except 1 deep infection were performed for ARMD. In addition, there are 2 more patients with suspected ARMD: one of which is scheduled for surgery, while the other has mild symptoms and refuses further treatment, leading to a total ARMD rate of 25%. LDHA THA sharing the same bearing as HR, adding the modular head introduced a problematic modular junction responsible for trunnionosis, and thus ARMD. Our macroscopic findings of blackened corroded debris at the head-neck junction at time of revision confirmed the failure mechanism. Higher failure rates were also reported with Durom LDH THA (28.9%) in comparison to Durom HR (2.3%) by Ridon et al. [25] after 10-year follow-up. Similarly, Konan et al. [23] after a mean follow-up of 9 years, reported a 2.1% revision rate in HR versus 12.5% in LDH THA. From these results we understand that the MoM LDH bearing was not the source of the failures. With an appropriate modular junction, it would provide low failure rate and then its stability benefits may become appealing.

Using a CoC LDH THA in 276 patients, we reported a revision rate of 1.4% after a mean follow-up of 67 months (min 48, max 79) [9]. The 4 revisions were unrelated to the modular junction or the bearing. Furthermore, we did not observe radiographic or clinical signs of ARMD. To indirectly assess the performance of the modular junction of this specific CoC LDH THA, we measured the Ti level in 57 unilateral cases after a mean follow up of 79 months [26]. We observed low mean Ti levels (mean 1.9, SD 0.53), suggesting good modular junction performance. If the encouraging results of these studies are confirmed in the long term then the use of CoC LDH THA, would be a favorable alternative to HR in the young active patient. In recent years, CoC or MoP HR were also introduced but femoral head necrosis and loosening would stay unresolved [8, 27, 28].

In our study no unrevised implant suffered a dislocation, highlighting the benefit of LDH THA and HR implants to reduce the dislocation and maximize range of motion. In a series of 1748 LDH THAs at a mean follow-up of 31 months, the dislocation rate was 0.05% [29]. In in a retrospective study comparing 559 conventional THAs and 248 LDH THAs at a mean follow-up of 5 years, the dislocation rate was 1.8% versus 0% respectively [30]. Similarly, in an RCT, we reported no dislocation rate in HR versus 3% in 28 mm THA [31]. Similarly, Pollard et al. in retrospective study, reported a dislocation rate of 7.4% among 54 THA compared to none in 54 HRs [1].

### Metal ion levels

Systemic Co and Cr ions levels are an indirect way to assess the in vivo bearing and/or modular junction performance. Although both implants in our study had the same bearing, Co ion levels were significantly higher in LDH THA, whereas no statistical difference in Cr could be found (Table 3). Moreover, the Co/Cr ratio was much higher in LDH THAs (1.9 vs 1.0). These differences can be explained by the poor performance of the modular junction of the Durom LDH THA. The wear and corrosion of the modular junction releases additional Co and Cr [32]. Part of the Cr wear particles precipitate locally, forming a black tartrate around the femoral neck, reducing the amount of Cr released systemically, explaining a higher whole blood Co/Cr ratio and the lack of measured difference in systemic Cr between groups [33]. Ridon et al. [25] reported 6-fold higher Co (*p* < 0.0001) and increased Cr (*p* < 0.0001) whole blood ion concentrations in patients with LDH THA compared to patients with HR, when comparing the results of the same acetabular component (Durom) after more than 10 years’ follow-up. Similarly, in a RCT evaluating the Durom system at 1 year postoperatively, patients with a LDH THA had 10-fold higher serum Co levels (*p* = 0.000) compared to patients with HR, and no difference for Cr levels [34]. Finally, metal ion levels were not statistically different over time in both groups, indicating that the unrevised LDH THA and HR prostheses continue to perform well in the long-term follow-up.

### Radiographic analysis

On radiographic evaluation at the last follow-up, all components were considered to be stable, except for 1 asymptomatic HR with a loose femoral component. Lucent lines on the femoral side were not seen in other HR, whereas proximal non-progressive femoral radiolucent lines observed in the LDH THA cases are known to be linked to normal long-term bone remodeling pattern of the CLS stem [35]. These results are reassuring as the remaining unrevised cases seem to be producing limited wear particles and should continue to do so in the future.

HR has been proposed as a great treatment option for the high expectations’ young patients. On the other hand, its indications may be limited in presence of secondary osteoarthritis associated with anatomical challenges, such as acetabular retroversion, hip dysplasia, femoral retroversion, Perthes, and pistol-grip deformity. LDH THA replacing the femoral head-neck pathological anatomy and offering a supraphysiologic head-neck ratio has the advantage of simplifying these complex cases [36].

## Conclusion

In this RCT comparing HR and LDH THA using the same bearing, after a mean of 14 years’ follow-up, LDH THA had a higher revision rate, linked to its modular junction wear and corrosion. However, the LDH THA group had still better PROMs compared to the HR group. LDH THA with a new modular junction design and/or ceramic bearing, obviating the trunnion issues would be a more appealing option than HR for our young and demanding patients.

## Data Availability

The datasets used and analyzed during the current study are available from the corresponding author on reasonable request.

## Abbreviations

ARMD: Adverse reaction to metal debris
CoC: Ceramic on ceramic
FJS: Forgotten joint score
HR: Hip resurfacing
LDH: Large diameter head
MoM: Metal on metal
MoP: Metal on polyethylene
PJP: Patient’s joint perception
PROMs: Patient reported outcome measures
THA: Total hip arthroplasty
